# [Retracted] Resveratrol pretreatment attenuates traumatic brain injury in rats by suppressing NLRP3 inflammasome activation via SIRT1

**DOI:** 10.3892/mmr.2026.13901

**Published:** 2026-05-05

**Authors:** Peng Zou, Xiaoxiao Liu, Gang Li, Yangang Wang

Mol Med Rep 17: 3212–3217, 2018; DOI: 10.3892/mmr.2017.8241

Following the publication of the above paper, it was drawn to the Editor's attention by a concerned reader that, in addition to the apparent duplication of control GAPDH western blots comparing Figs. 1A and 2C, the SIRT1 western blot data featured in Fig. 5A on p. 3215 had already been published previously in an article written entirely by different authors in different departments of the same research institute in the journal *Mediators of Inflammation*.

Owing to the fact that the contentious data in the above article had already been published prior to its submission to *Molecular Medicine Reports*, the Editor has decided that this paper should be retracted from the Journal. The authors were asked for an explanation to account for these concerns, but the Editorial Office did not receive a reply. The Editor apologizes to the readership for any inconvenience caused.

